# High-density via RRAM cell with multi-level setting by current compliance circuits

**DOI:** 10.1186/s11671-023-03881-x

**Published:** 2024-03-25

**Authors:** Yu-Cheng Hsieh, Yu-Cheng Lin, Yao-Hung Huang, Yu-Der Chih, Jonathan Chang, Chrong-Jung Lin, Ya-Chin King

**Affiliations:** 1 Institute of Electronics Engineering, National Tsing Hua University, Hsinchu, Taiwan; 2 Design Technology Platform, Taiwan Semiconductor Manufacturing Company, Hsinchu, Taiwan

**Keywords:** RRAM, MLC, NVM

## Abstract

In this work, multi-level storage in the via RRAM has been first time reported and demonstrated with the standard FinFET CMOS logic process. Multi-level states in via RRAM are achieved by controlling the current compliance during set operations. The new current compliance setting circuits are proposed to ensure stable resistance control when one considers cells under the process variation effect. The improved stability and tightened distributions on its multi-level states on via RRAM have been successfully demonstrated.

## Introduction

In past decades, semiconductor memories are widely used in many fields. Meeting the requirement of various electronic products, the memory density on integrated circuits has been pushed aggressively higher over the years. Volatile and nonvolatile memories are two types of memories commonly used in computing systems. DRAM (dynamic random access memory) [1] and SRAM (static random access memory) [2] are volatile memories, whose data will be lost when power is off. Flash memory is the most successful nonvolatile memories in existing market, which is used in many portable electronics. Flash memory is conventionally based on floating gate structures, and the stored data depend on the amount of the charge stored in the floating gates [3, 4]. To overcome the physical limit of the floating-gate-based memory technologies, various NVM solutions have been proposed and developed. Emerging NVMs addresses the scaling challenges of embedded flash memories [5]. It improves its compatibility toward scaled CMOS technology, density as well as operation speed. Therefore, the emerging NVMs become one of the most potential candidates to replace the existing embedded NVM solutions [6].

Resistive random access memories (RRAM) become one of the emerging nonvolatile memories because its many advantages, such as low operative voltage, high-speed program, low power consumption, low cost and compatible with CMOS logic process [7]. A RRAM device typically consists of a simple metal–insulator–metal (MIM) structure. The insulator material between the two electrodes is composed of transition metal oxide (TMO) and becomes the resistive switching layer, where data are stored. Previous studies have revealed that this resistive switching layer can change between the low-resistance state (LRS) and the high-resistance state (HRS) by giving the proper voltage or current [8]. For a typical 1T1R cell, the density of a memory array depends on line-to-line spacing limits by the critical dimension of the technology. To further increase the data density in a memory technology, multi-level storage is often introduced [9, 10].

In this work, the multi-level operation of via RRAM is achieved by the select transistor control. However, the select transistor current varies from cell to cell, which prevents precise compliance current control. Here, new current compliance methods are proposed to ensure stable current during set operation of realizing MLC in via RRAM. These circuits are less susceptible to process variation effect, leading to more stable resistive states; namely, the resistance distributions can be effectively tightened. The new operation scheme helps to achieve stable multi-level storage in full-logic compatible via RRAM cell.

## Circuit and operation method

**Fig. 1:**
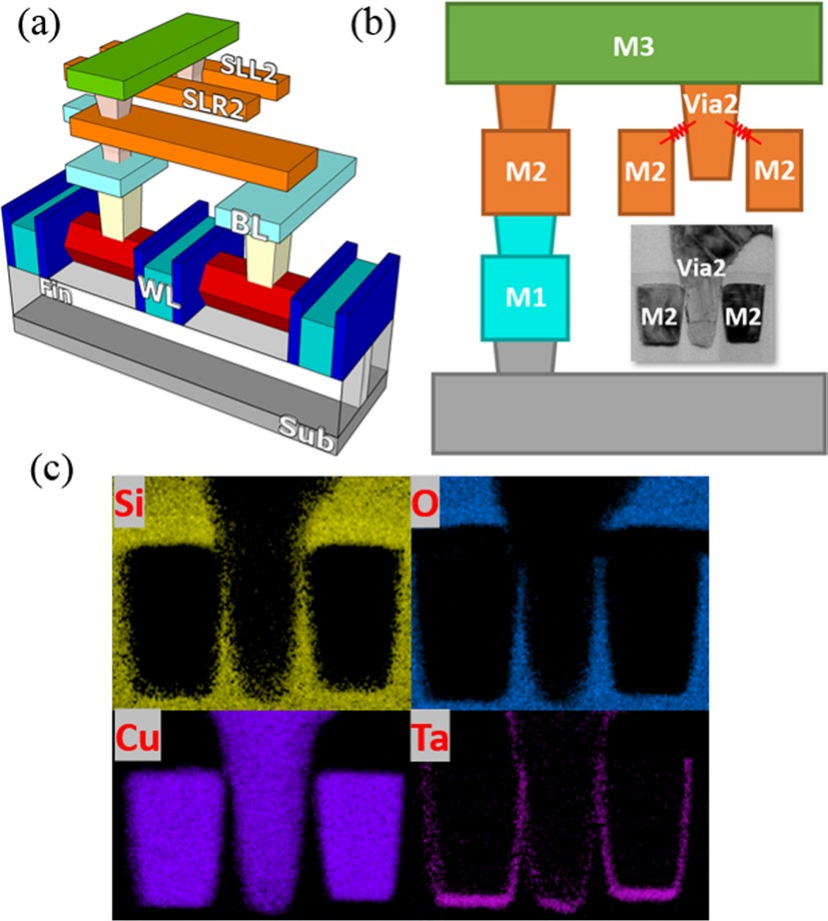
**a** Illustration of the via RRAM cell implemented by the 16-nm FinFET CMOS logic process and **b** TEM cross-sectional view of a 1T2R cell which includes one select transistor and a pair of via RRAM on both sides of recessed via between metals. **c** EDS cross-sectional view of the via RRAM MIM structure, including the electrodes and transition metal oxide layer

The via RRAM cells were proposed in a previous study [11] and implemented using the FinFET CMOS logic process for the subsequent experiments. These cells consist of via RRAM formed between each metal layer and via, which can be stacks in 3D array. This study primarily focuses on the multi-level cell (MLC) characteristics in the first layer, with the aim of applying MLC features to achieve high-density 3D memory array in the future. The illustration of the via RRAM single cell is shown in Fig. 1a. The single one-transistor–two-resistor (1T2R) structure consists of one transistor and a via RRAM pair. The FinFET serves as the select transistor, controlling its corresponding forming/set/reset processes. In an array, the gate of the select transistor is connected to the word line, WL, and its source is connected to the bit line, BL. Figure 1b shows the transmission electron microscopy (TEM) photograph and the cross-sectional view of the via RRAM samples along the WL direction. The via RRAM is composed of the via and the metal on both sides of the via, given two storage nodes per via [12].

**Fig. 2:**
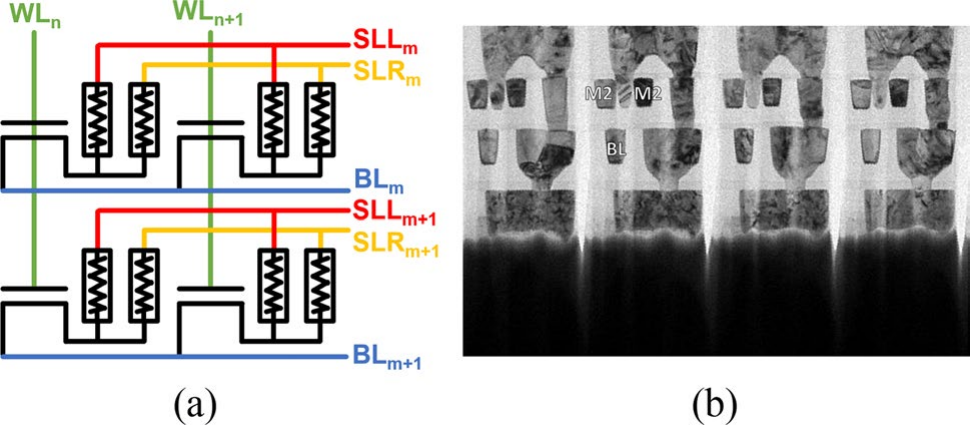
**a** Circuit schematic of the 2 × 2 via RRAM array and **b** TEM picture of the via RRAM array along WL directions

Figure 1c shows the EDS analysis graph, illustrating the main materials used in the via RRAM. The via acts as one electrode in the MIM structure, while the metal layer serves as the other electrode. The top electrodes are connected to two separate source lines, SLL and SLR, respectively. The TMO layer of the via RRAM between the via and the metal electrodes is composed of TaO_x_ and SiO_x_ [13], and this TMO layer serves as the resistive switching film, with a thickness of approximately 14 nm. Moreover, the thickness of the TMO layer is designed by the clearance between the metal and via in the layout. The Ta in the TMO layer originates from the copper barrier layer, while SiO_x_ is the material of the interlayer dielectric (ILD) layer, which accounts for approximately 8 nm in the overall TMO thickness. Additionally, the thickness of the TMO layer is positively correlated with the voltage required for the subsequent forming operation. For a more compact cell, via RRAM can be placed on top of the transistor; hence, the density of the via RRAM array can be further enhanced. Figure 2a shows the 2 × 2 via RRAM array structure, the WL is perpendicular to the transistor channel direction, and the BL and the SL are perpendicular to the WL direction. For the set operation of the select cells, the WL voltage is given to the select transistor for preventing overset. For the unselected cells, BL is kept floating to decrease the disturbance to the unselected cells. The TEM of the via RRAM array structure is shown in Fig. 2b, and two via RRAM are placed above a single FinFET transistor; hence, its packing density is further increased.

**Fig. 3:**
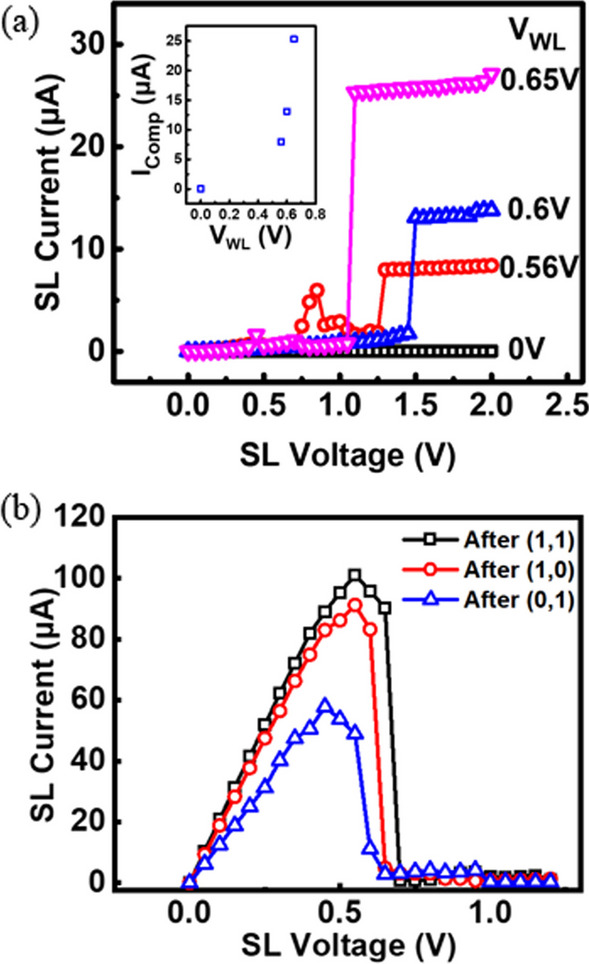
**a** Set characteristics of the via RRAM in reaching four different resistive states by the WL voltage control. **b** The reset DC characteristic graph is obtained after corresponding set operations by different WL voltage control

In the previous study, it has been reported that the resistive levels of a RRAM depend strongly on current compliance levels [14–16]. Based on previous results, we expected that different resistive levels can be achieved by proper WL voltage control. Figure 3a shows the DC set characteristics of achieving multi-level states in via RRAM devices. The initial state of RRAM is in a high-resistance state. The initial forming operation is needed to convert the RRAM into a LRS. The forming operation has significant impact on the distributions of subsequent conductive filaments (CFs), so a WL voltage of 0.65 V and a SL voltage of 2 V are used in forming to reach the lowest resistance level to ensure large enough window to accommodate the other states. Data reveal that four distinct via RRAM states can be readily reached by proper WL voltage control during set operations. To keep a cell in the HRS, WL voltage of 0 V is applied. Since the select transistor is off, the voltage between top and bottom electrodes of the via RRAM is very small, so that the RRAM states is not disturbed and remain in HRS. The subsequent resistive levels depend on the WL voltage, a larger WL voltage during set results in lower-resistance states. These characteristics can be explained by different forms of CFs in the resistive oxide layer. When the via RRAM is set by a larger WL voltage of the select transistor, as its state charges from HRS to LRS, the clamping current increases with V_WL_. During the set operation, a sufficiently large electric field is applied between two electrodes to separate oxygen negative ions from positively charged the vacancies, where the migration direction of negative ions is opposite to that of the applied electric field [17]. The conductive filaments (CFs) in the TMO layers and the distribution of the remaining vacancies determine the resulting resistance levels. Higher compliance current is known to increase the probability of oxygen vacancies generation, and the elevated level of oxygen vacancies is left in transition metal layer [18]. Therefore, more densely distributed CFs are formed because of higher compliance current during set operation. Consequently, lower-resistance state is obtained under such set operations [7, 19].

Figure 3b illustrates the DC reset characteristics, corresponding to the transformation of the LRS RRAM to HRS RRAM in three set operations shown in Fig. 3a. Compliance currents can be controlled by different WLs during the set operations and leads to difference in the LRS states. Larger compliance currents during set lead to a higher reset current to accomplish switching the states back to HRS.

**Fig. 4:**
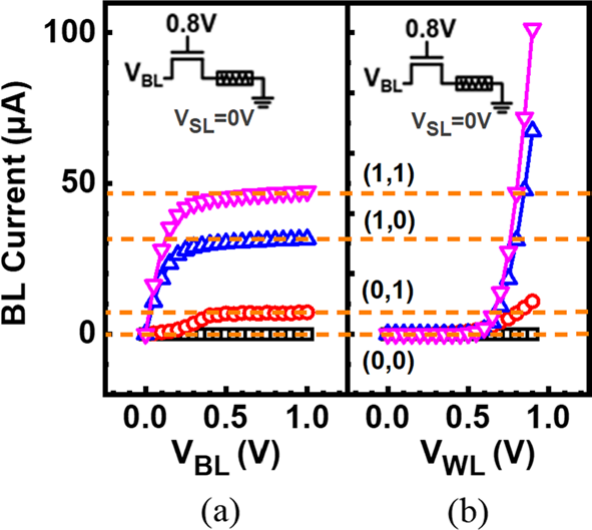
Read characteristics of multi-level states **a** under a fixed BL voltage of 0.8 V and **b** under a fixed WL voltage of 0.8 V. The proper read voltage is V_WL_ = 0.8 V and V_BL_ = 0.8 V

To determine a suitable read condition, the WL and the BL voltage must be selected to distinguish different resistive levels with a good margin, while avoiding altering the stored data. To avoid read disturb, reverse read operation with voltage applied to BL is adapted for read. Figure 4 shows the read characteristics under a fixed BL or WL voltage. To test the response of RRAM cells under different states, a cell is given a fixed WL voltage of 0.8 V, while BL ramps from 0 to 1 V. The read current gradually saturates while the BL voltage increases, and the select transistor can prevent the via RRAM from changing the resistance state accidentally. Subsequently, a cell is given a fixed BL voltage, and WL sweeps to 0.9 V. Shown as the read characteristics, it reveals that each resistance state will be distinguished when WL is given a voltage larger than 0.8 V. However, higher WL voltage will cause the disturbance of the originally stored states. Here the read condition is set as V_WL_ = 0.8 V and V_BL_ = 0.8 V, while 4 states storing 2 bits of (1,1), (1,0), (0,1) and (0,0) can be obtained.

**Fig. 5:**
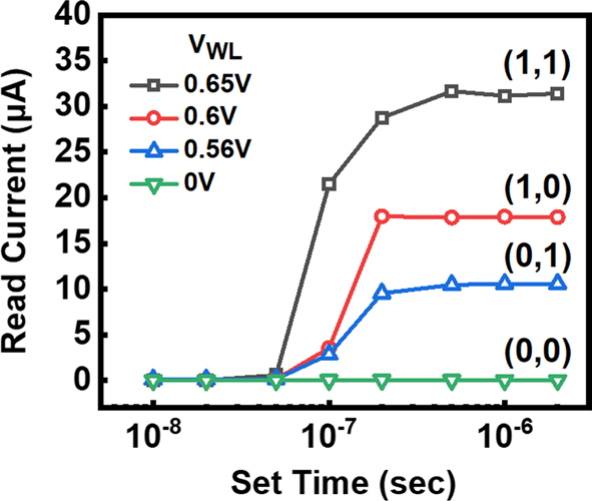
The time-to-set characteristics of the four states in the via RRAM were measured by applying AC pulse signals while keeping the SL voltage fixed. This measurement aimed to evaluate the operational speed at different WL voltages. It was observed that the higher WL voltage leads to faster set operations

**Fig. 6:**
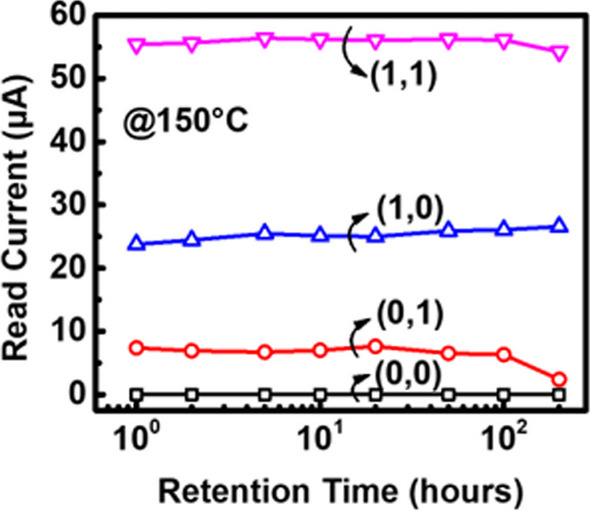
Stability of the multi-level via RRAM tested under high-temperature bake are demonstrated for over 200 h

The via RRAM also features high-speed programming and low operation voltage. Figure 5 illustrates the AC time-to-set characteristics of the four states in the via RRAM. The WL voltages were fixed at 0.65 V, 0.6 V, 0.56 V and 0 V, respectively, while the AC pulse signals were applied to the SL terminal with a fixed SL voltage of 2.5 V. The operation time was confirmed by applying accumulating pulses to the cells. It is found that different WL voltages affect the magnitude of the limiting current of the transistor after the RRAM change into LRS, and larger WL voltages result in shorter set times with insignificant difference. Regarding the SL voltage, considering cell-to-cell variation, we verified that via RRAM exhibits slower operation speed at lower SL voltages, and some RRAM cells are unable to complete the set operation successfully. At an SL voltage of 1.5 V, the measured operation speed was approximately 200us. However, through experiments, we confirmed that increasing the SL voltage to 2.5 V allows via RRAM to achieve operation speeds as fast as 200 ns, and most cells can complete the operation successfully. Therefore, an SL voltage of 2.5 V is more suitable for the set operation of via RRAM. Overall, the set operations to all four states can be achieved within 200 ns. To evaluate data retention of MLC via RRAM at different states, devices are baked under 150℃. Figure 6 shows the read current levels of the via RRAM under high-temperature bake for 200 h. Data show that the 4 different states remain stable during the test period, suggesting good data retention capability.

## Results and discussions

**Fig. 7:**
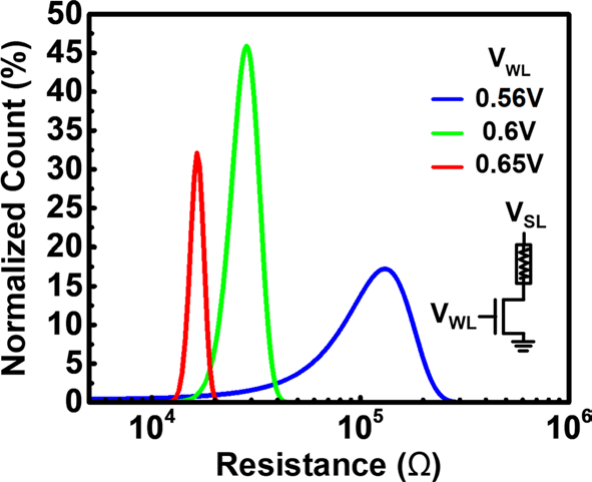
Distribution of the multi-level via RRAM obtained by the single MOS WL voltage compliance during set operation

To study process variation effect of via RRAM control, Fig. 7 shows the resistance distribution of the multi-level via RRAM set by different WL voltage during set operation. It reveals that the resistance windows can be obtained by the proper WL voltage control. Data suggest the overlap between different resistance states when multiple samples are measured in a memory array as a result of process variation. The overlap resistance states may cause read errors. The compliance current variation is mostly caused by threshold voltage variation from cell to cell. The unstable compliance current leads to the large distribution of the resistance states. Hence, the overlap between resistance states occurs, as shown in Fig. 7.

**Fig. 8:**
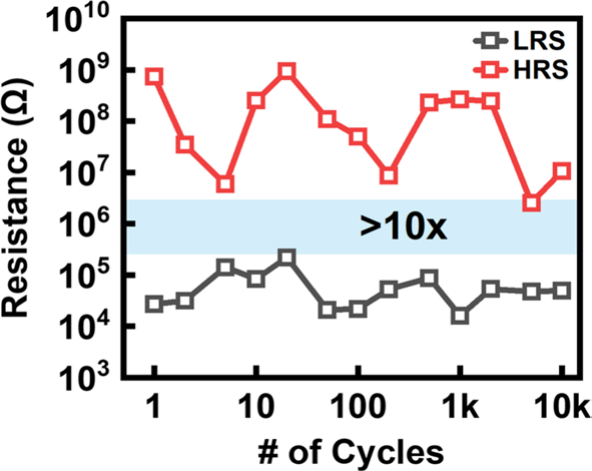
Endurance characteristics of M2/via2 RRAM with 10,000 cycles

In addition, the set operation was performed by fixing the WL voltage at 0.65 V, and the endurance test conducted under this condition verifies that the via RRAM can achieve > 10,000 set/reset cycles with a reading window greater than 10X as shown in Fig. 8. Similarly, as mentioned above, despite fixing the WL voltage and using a single MOS for current compliance, the via RRAM still exhibits variations in LRS between multiple operations during cycling. Therefore, a more precise compliance current control circuit is a must for multiple level cell operations.

**Fig. 9:**
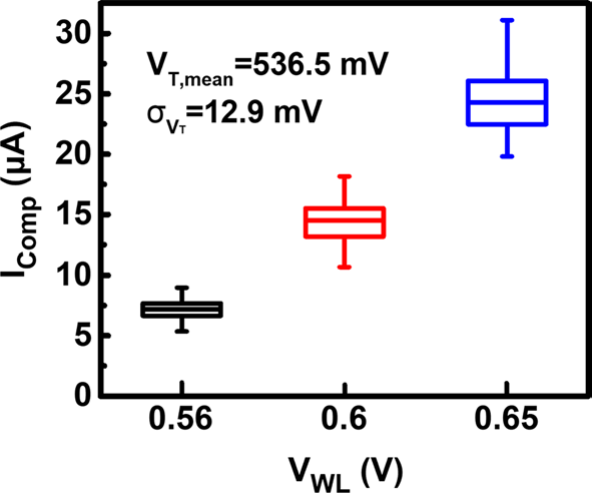
Current distribution of a single MOS under three different WL voltages. Variation in transistor’s threshold voltage can lead to the large variation of compliance current during set. Data show that the higher the WL voltage is applied, the wider the compliance current spread is

As above, under a fixed WL voltage, the compliance current set by a single transistor control may vary widely, which leads to poor control of the RRAM resistance states. Figure 9 shows the measured compliance current of the single select transistor under different WL voltages. It reveals that there is a large variation under a fixed WL voltage. The compliance current variation results from the threshold voltage variation of the select transistors. To improve the compliance current control during set operations of via RRAMs, two circuits are proposed to reduce the processing variation effects on the final MLC states.

**Fig. 10:**
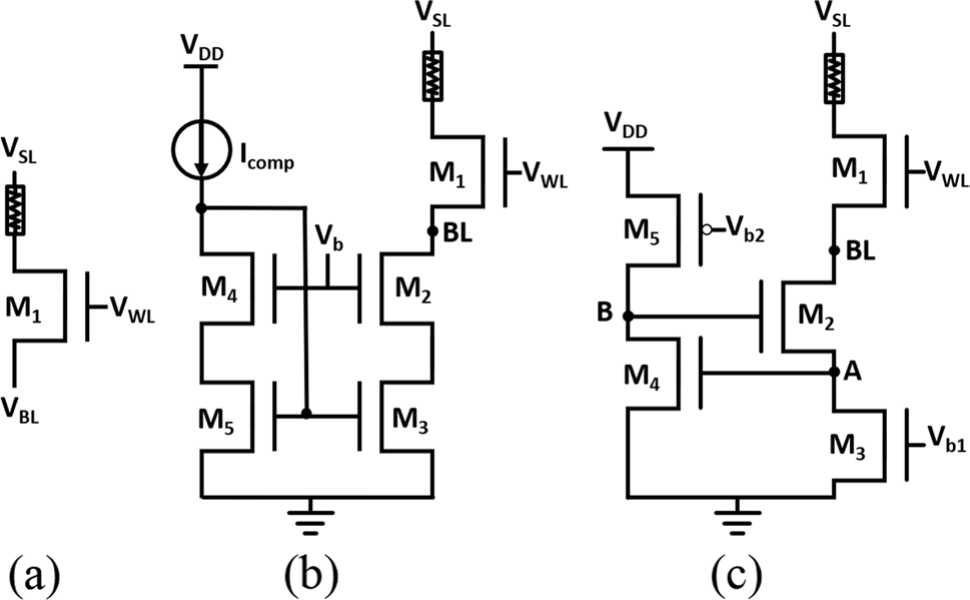
Compliance circuits: **a** single MOS, **b** current compliance current mirror, **c** negative feedback current compliance circuit

Three ways of controlling the set compliance current are compared in Fig. 10. First, a single transistor with WL control is used as the reference in Fig. 10a. A set circuit with a current mirror illustrated in Fig. 10b is expected to further assert more control over the compliance current levels [20–23]. Figure 10c shows the negative feedback current compliance circuit [24, 25]. In the conventional 1T1R storage, the select transistors provide select/protect function in the via RRAM cell. By adding these two column-shared set circuits, compliance current circuits are connected to BLs node in via RRAM array through a WL control switch. While setting the selected via RRAM, the WL of the FinFET transistor is given V_DD_ to select the via RRAM and given a set voltage on the SL. Thus, the set voltage can mostly be dropped on the via RRAM. As the resistive states changes from HRS to LRS, the current of the via RRAM increases suddenly, but these set circuits are expected to protect the via RRAM from being overset. Moreover, the set circuits will also provide stable compliance current, reducing the variation of each resistance state.

**Fig. 11:**
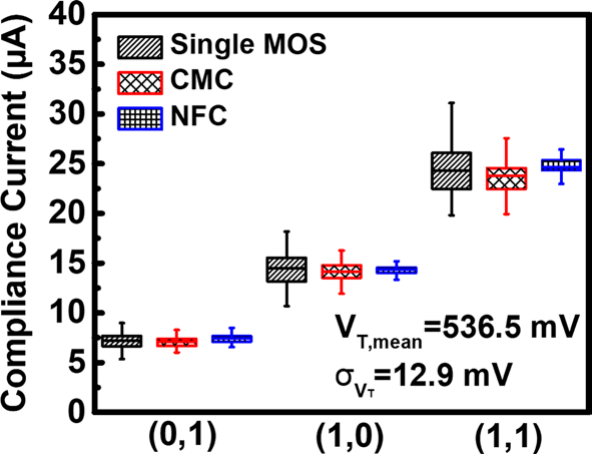
Comparison of current compliance by different compliance circuits, the negative feedback current compliance circuit is better than the single MOS and the current compliance current mirror

**Fig. 12:**
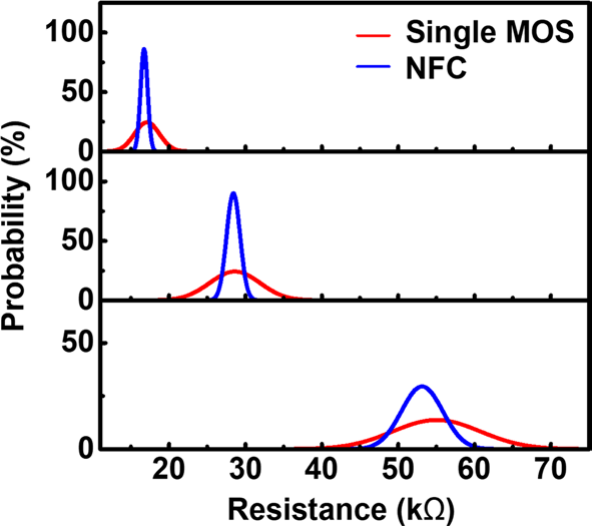
Comparison of resistance distribution of the via RRAM programmed by the single MOS and the negative feedback current compliance circuit

The current variations during set to different states by the three different methods are compared in Fig. 11. Data show that the negative feedback circuit has the best stability in its set current control. It implies that set by the negative feedback current compliance circuit will result in less variation of the resistive states. Figure 12 shows the comparison of the best case and the worst case, respectively. The negative feedback current compliance circuits achieve more stable resistive states compared to the select transistors. The simulated result shows that the negative feedback circuits are less susceptible to process variation effect. For cells with higher threshold voltage of the select transistors, compliance current level is expected to reduce as overdrive voltage on M_1_ decreases. In the negative feedback circuit, the decreasing voltage on node A increases M_4_ equivalent resistance. Then, node B is pulled up, and M_2_ equivalent resistance is reduced. As M_2_ equivalent resistance becomes smaller, BL voltage drops, and the overdrive voltage on M_1_ will remain relatively constant. Finally, the compliance current level of NFC is less susceptible to process variation effect, as demonstrated by the results in Fig. 12.

## Conclusions

In this study, the multi-level via RRAM with standard 16-nm FinFET CMOS logic process is proposed. First, the multi-level via RRAM is achieved by the current compliance of a single transistor. Unstable compliance current leads to the overlap between states when process variations are considered. To avoid this problem, the new current compliance setting circuits are presented, which provide a more stable compliance current, hence tightening the distribution of the resistance states successfully.

## Data Availability

The datasets generated during and/or analyzed during the current study are not publicly available due to the policy of our cooperative research company but are available from the corresponding author on reasonable request.
